# The Role of Exosomes and Their Applications in Cancer

**DOI:** 10.3390/ijms222212204

**Published:** 2021-11-11

**Authors:** Yuju Zhou, Ying Zhang, Huan Gong, Siqi Luo, Yan Cui

**Affiliations:** School of Pharmacy, Shenyang Pharmaceutical University, Shenyang 110016, China; zyj3488398542@126.com (Y.Z.); yingzhang0713@126.com (Y.Z.); ghuan23@163.com (H.G.); lsq1362543554@163.com (S.L.)

**Keywords:** exosomes, tumor microenvironment, cancers, applications

## Abstract

Exosomes are very small extracellular vesicles secreted by multiple cell types and are extensively distributed in various biological fluids. Recent research indicated that exosomes can participate in regulating the tumor microenvironment and impacting tumor proliferation and progression. Due to the extensive enrollment in cancer development, exosomes have become a focus of the search for a new therapeutic method for cancer. Exosomes can be utilized for the therapeutic delivery of small molecules, proteins and RNAs to target cancer cells with a high efficiency. Exosome-carried proteins, lipids and nucleic acids are being tested as promising biomarkers for cancer diagnosis and prognosis, even as potential treatment targets for cancer. Moreover, different sources of exosomes exhibit multiple performances in cancer applications. In this review, we elaborate on the specific mechanism by which exosomes affect the communication between tumors and the microenvironment and state the therapeutic and diagnostic applications of exosomes in cancers.

## 1. Introduction

Cancer has become a major threat to human health, with increasing mortality around the world [1]. The tumor microenvironment (TME) plays a vital role in cancer onset and progression through information communication, which is conducive to tumor cell proliferation, angiogenesis and distant metastasis [2]. Cell communication is also critical in specific pathological processes. Indeed, cancer cells need to cross-talk to each other, normal cells, and immune system to survive, proliferate and metastasize [3]. Exosomes have been extensively studied because of their fascinating biological roles in cell-to-cell communication. Communication between the tumor and exosomes leads to modifications of the TME favoring tumor growth, survival, immune-escape and invasion [4].

As one of the most impactful components in the TME, exosomes have been demonstrated to possess specialized functions in tumor initiation, progression, metastasis, angiogenesis and drug resistance [3]. Through a series of natural cytokines and growth factors carried by exosomes, immune cells and lymphoid components of the TME, such as B and T lymphocytes, natural killer cells, and macrophages, could be activated or inhibited by exosome-related grow factors and cytokines, resulting in immunosuppression and tumor progression [5,6]. In addition, the application of exosomes in drug delivery have gained widespread recognition [7,8]. In fact, their suitability as ideal drug delivery vehicles is due to the capacity of exosomes to overcome limitations such as poor bioavailability, non-targeted cytotoxicity and poor immunogenicity of drug carriers [9,10]. They have been designed to delivery all kinds of drugs for cancer therapy in animal model such as small molecules, nucleic acids and proteins. Besides, exosomes are released by various cell types under both normal and pathological conditions [11]. The cell-specific proteins and genetic materials in exosomes are capable of reflecting their cell origin and physiological status, which could be explored as preclinical biomarkers in many types of cancers, such as lung cancer, hepatocellular carcinoma, pancreatic cancer, colorectal cancer, melanoma, breast cancer, prostate cancer, ovarian cancer, glioblastoma and nasopharyngeal carcinoma [11,12,13,14,15,16,17]. Additionally, alternative therapeutic strategies, such as the inhibition of exosome production, as well as the blockage of the uptake of exosomes to specific receptors, have been proposed as novel cancer interventions [18]. All together, the widespread application suggests that various potential therapeutic strategies by intercepting the biogenesis, secretion, or uptake of tumor derived exosomes are promising means for the development of anticancer therapies in the future [19,20].

Mounting evidence shows that exosomes can be released from different types of cells, such as macrophages, dendritic cells, tumor cells, mesenchymal stem cells, epithelial cells, mast cells, endothelial progenitor cells, platelets, lymphocytes and fibroblasts. Exosomes derived from different cells exhibit different characteristics and functions [21,22]. Among these cells of origin, MSCs are the most frequent source of exosomes for cancer treatment. They are used in many types of cancers in labs for animal models, including melanoma, breast cancer and glioma [18,23]. Apart from MSCs-derived exosomes, exosomes from cancer cells and milk are also used in the treatment of various diseases in animals [24]. Regardless of the origin of exosomes, they can all mediate the process of tumor proliferation or inhibition through various signal pathways [25].

This review aims to present a comprehensive and critical overview on the recent progresses of exosomes in cancer, including the connections between exosomes and cancer, the applications of exosomes in cancer and the functions of exosomes from different sources. Recent advances in exosomes will be beneficial to realize clinical applications for cancers someday.

## 2. The Role of Exosomes in the TME

**Figure 1:**
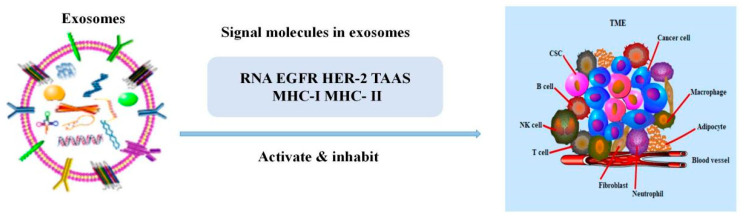
The relationship between exosomes and the TME. The signal molecules carried by exosomes could activate or inhibit immune cells to mediate the growth of tumor cells.

It is well known that the TME is highly heterogeneous, including tumor cells, various stromal cells and the microenvironment in which they reside. In tumor development and metastasis, tumor cells do not act alone, but interact with the whole tumor microenvironment [26,27]. Accumulating evidence indicates that exosomes are crucial in the regulation of the TME [26,28], in fact, they can alter the expression of the extracellular matrix, stromal cells (CAFs, CSCs, MSCs) and immune cells in the TME [29] (Figure 1). Among them, changing the expression of immune cells is the main way for exosomes to regulate the TME. Exosomes can further regulate tumorigenesis and metastasis by regulating the interaction between T lymphocytes, B lymphocytes, macrophages, natural killer (NK) cells and the TME (Table 1). [30,31].

### 2.1. T Cells

T cells are one of the most important cells in regulating the immune system. They mediate key immune responses in the context of infection, cancer and autoimmune diseases. In recent years, the tight connection between exosomes and T cells has gradually received extensive attention. Tumor-derived exosomes (TEXs) can protect T cells from cancer cell-mediated apoptosis by secreting natural cytokines and growth factors, ultimately activating the immune system and inhibiting tumor initiation and metastasis [51]. For example, tumor-derived exosomes were able to secrete tumor associated antigens (TAAs), various growth factor receptors (EGFR or HER-2), and major histocompatibility complexes (MHC-I and MHC-II) [52]. Through the direct or cross presentation of antigen-presenting cells (APCs), these substances were delivered to the corresponding location to activate CD8^+^ and CD4^+^ T cells. Finally, established tumors were directly suppressed or eliminated [53].

Cytotoxic T cells (CTLs), also known as killer T cells, are important members of the adaptive immune system [32]. Exosomes can also induce the generation of cytotoxic T cells [33]. For example, malignant glioma-derived exosomes contain antigen-presenting molecules (MHC-I, heat shock protein 70), tumor antigens (MAGE-1) and adhesion molecules (ICAM-1). These substances could activate CD8^+^ T cells into CTLs that can kill glioma cells [33,34].

Conversely, exosomes interestingly play an equally contrasting role of immunosuppression in cancer. Tumor-derived exosomes express key immunosuppressive cytokines and signaling molecules (FasL, TRAIL, PD-L1) which could induce cancer cells’ escape from immune recognition and T cells apoptosis. Characterization studies revealed that in melanoma cells, melanosome-positive MVBs contain exosomes which could express bioactive FasL and induce T apoptosis to promote tumor growth [35,36,54]. PD-L1 is a membrane bound-ligand which can be upregulated in inflammatory and neoplastic contexts [55]. Tumor exosomal PD-L1 could bind its receptor on immune T cells, leading to the dephosphorylation of the T cell receptor and its co-receptor CD28, further inhibiting the activation of T cells [37,56]. Moreover, Czystowska et al. showed that phosphatase and tensin homolog resided in TEX and have the ability to activate CD8^+^ T cells, resulting in T cells apoptosis and tumorigenesis [31,57].

Regulatory T cells (Tregs) are a kind of critical immune regulatory cell in the cancer-immune tolerance. The cellular immune system in the TME and TEXs can directly support Tregs expansion and their suppressive functions [31,58]. There was a report indicating that TEXs induced a transition of CD4^+^ CD25^neg^ T cells to CD4+ CD25^high^ Foxp3^+^ Tregs via upregulating growth factor TGF-β1 and IL-10. Eventually, the tumor surpassed immune escape through the immunosuppressive function of Tregs [38].

### 2.2. B Cells

B lymphocytes are mainly involved in initiative immunity processes and have multiple functions in tumor immunity. They can secrete immunoglobulins (antibodies), present antigens, provide costimulatory signals and release cytokines to support or suppress tumor immunity [59,60]. It has been confirmed that exosomes can mediate the above pathways to regulate the immune effects of B cells against tumors.

In vitro studies showed that exosomes secreted by mycoplasma-infected BMSCs were capable of inducing polyclonal activation of all B cell subsets and promoting immunoglobulin activation [61]. Yang et al. demonstrated that exosomes released by mycoplasma-infected tumor cells specifically activated inhibitory B cells and induced the production of pro-inflammatory factors IFN-c and anti-inflammatory IL10 [39]. Furthermore, exosomes harbored B cells targets in pancreatic adenocarcinoma (PDAC) and played a revulsive role in complement-mediated cytotoxicity. They induced the splenic B cell zone to capture and transport antigens in complement-dependent manner, resulting in activation of B and T cells [40].

Besides, exosomes can regulate the activity of B cells by secreting or carrying other substances such as proteins [62]. It was found that exosomes generated a malignancy resistance effect by mediating the expression of the transmembrane phosphoprotein CD20 on mature B cells as well as the vast majority of B-NHL cells [40]. Helen Vallhov et al. proved that gp350 in chronic B lymphocytic leukemia acted not only as a chemotactic molecule for exosomes but also as an antigen binding to CD154 receptor in B cells tumor immunity [63], ultimately rendering B cells immunogenic [41], which offered new possibilities of immunotherapy for other malignancies associated with B cells.

Regulatory B cells (Bregs) are major immunosuppressive B cells. It has been reported that exosomes modulated immune responses by promoting the generation of Bregs [39]. Capello et al. proved that exosomes from esophageal squamous cell carcinoma inhibited the proliferation of B cells, inducing the expansion of interleukin-10^+^ Bregs (B10) and (PD)-1 Breg cells [64]. Li et al. isolated tumor-derived microvesicles from esophageal carcinoma tissue (ECA) and demonstrated that ECA-derived exosomes could carry MMP9 to promote the differentiation of naive B cells into Bregs [42]. Furthermore, Ye et al. found that human hepatocellular carcinoma (HCC) exosome-derived HMGB1 activated B cells and promoted the proliferation of TIM-1^+^ Breg cells via TLR 2/4 and MAPK signaling pathways [65].

### 2.3. NK Cells

NK cells, a kind of significant immune cell in human body, participate in resisting viral tumors or killing tumor cells through multiple pathways. Unlike T and B lymphocytes, their cytotoxic activities are neither restricted with the major MHC nor dependent on antibodies. The response mechanism of NK cells is mainly determined by a complex interaction of inhibitory and activated receptors [66]. Early studies reported that there was a certain relationship between exosomes and NK cells [67]. In fact, TEXs inhibited tumor immunity through interference with NK cells. On the one hand, exosomes affect the number of NK cells through the regulation of NK cell receptors expression. These receptors are broadly classified into killer immunoglobulin receptors (KIRs), natural cytotoxicity receptors (NCRs) and C-type lectin-like receptors [68]. After encountering TEXs, the expression of NK cells’ activating receptors (NKP30, NKP46, NKG2C, NKG2D) are downregulated, thereby inhibiting NK cells’ proliferation [69].

In acute myelogenous leukaemia (AML) patients, exosomes were enriched in CD34, CD33 and CD117. The expression of the NKG2D receptor was downregulated and Smad phosphorylation was induced in NK cells [67], leading to tumor occurrence. The HSP-bearing exosomes, secreted by human hepatocellular carcinoma cells, could upregulate the expression of the inhibitory receptor CD94 and downregulate the expression of the activating receptors CD69, NKG2D and NKP44 on NK cells, which enhanced the proliferation of liver cancer cells [69].

On the other hand, exosomes alter the immune activity of NK cells by carrying or releasing cytokines and other substances, including IL-15Rα, HSP 70 [70], TGF-β, MICA008, STAT5, JAK3, cyclind3, perforin, etc. Several studies have shown that TGF-β1 carried by TEXs impaired NK cells cytotoxicity and lowered NKG2D expression in the NK cells of acute myeloid leukemia patients [43]. In addition to TGF-β1, Berchem et al. proved exosomal miR-23a from lung carcinoma cells could suppress the function of NK cells [44]. Significantly, interleukin-2 (IL-2) was essential for promoting the survival, proliferation and functional differentiation of NK lymphocytes [71]. However, Clayton et al. proved that tumor exosomes directly inhibited the killing ability of NK cells by impairing the proliferative response to IL-2 [45].

### 2.4. Macrophages

Macrophages are the major immune cells in the TME, which mediate the regulation of inflammation [72]. They have two broad phenotypes, M1 and M2. M1 macrophages can kill tumor cells, whereas M2 macrophages promote tumor growth and metastasis [29,72]. Extensive studies have revealed that exosomes can influence M1 and M2 macrophage polarization to regulate the TME [73].

Tumor-associated macrophages (TAMs) are one of the M2-like macrophages [74]. They promote angiogenesis, tumor growth and metastasis by secreting proangiogenic factors and cytokines [74]. Some studies have shown that exosomes acted as substance transporters in the process of macrophage phenotype polarization [46]. For example, in epigallocatechin gallate (EGCG) treated breast cancer cells, miR-16 was delivered to macrophages via exosomes after being upregulated by EGCG, and finally, macrophages were transformed into M2 type [46]. Similarly, exosomes in rectal cell carcinoma promoted the expression of M2 markers by transporting miR-203, ultimately differentiating monocytes into M2 type macrophages with immunosuppression [47,75]. Besides, exosomes can also promote macrophage polarization toward M2 type by assisting in the activation of signaling pathways. For instance, breast-cancer-derived exosomes altered macrophage polarization to M2 phase via glycoprotein 130/STAT3 signaling. Specifically, exosomes isolated from breast cancer cells were enriched with glycoprotein 130, which was transferred to macrophages to induce M2 polarization [48].

Although M1 macrophages are less studied than M2 macrophages, exosomes can still influence macrophages polarization toward M1 type. A previous study showed that exosomes derived from squamous cell carcinoma cells carried and released THBS1, a protein with strong anti-inflammatory properties, resulting in macrophages polarization to M1 phenotype [49]. Moreover, miR-21 derived from colorectal cancer exosomes could drive macrophage polarization towards an M1 type secreting IL-6 [50].

**Figure 2:**
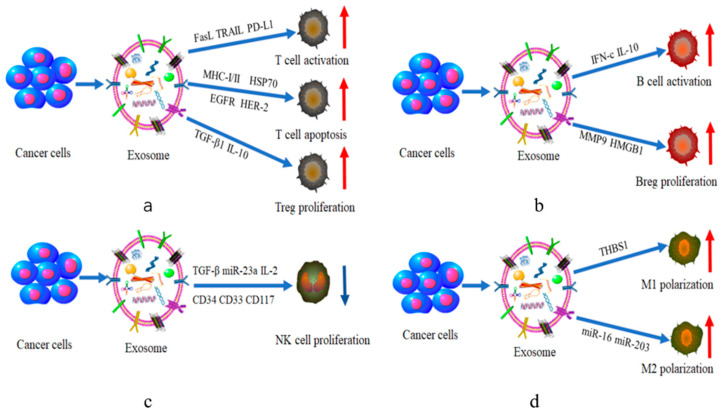
The effect of tumor-derived exosomes on immune cells. (**a**–**d**) respectively show the regulatory effects of various signal molecules of tumor-derived exosomes on the proliferation and differentiation of T cells, B cells, NK cells and macrophages. (MHC-I(II), major histocompatibility complexes; HSP70, heat shock protein70; EGFR HER-2, growth factor receptors; FasL TRAIL PD-L1, tumor necrosis factor–related apoptosis-inducing ligand; TGF-β, transforming growth factor β; IL-10, Interleukin-10TGF-β, transforming growth factor β; CD34 CD33 CD117, NK cells receptors; IFN-c, pro-inflammatory factors; IL-10, anti-inflammatory factors; MMP9, matrix metalloproteinase 9; HMGB1, an evolutionarily conserved DNA-binding nuclear protein; THBS 1, a protein with strong anti-inflammatory properties).

The above evidence prove that tumor-derived exosomes could induce the growth, proliferation and transformation of immune cells through various signal molecules, and this specific effect is shown in (Figure 2).

## 3. The Application of Exosomes in Cancers Therapy

### 3.1. As Useful Carriers in Drug Delivery System

Up to now, there have been many types of drug delivery systems (DDS) for oncotherapy, including synthetic polymers, liposomes, micelles, super magnetic particles, proteins and recombinant viral vectors [76,77,78,79]. However, most of the nanocarriers encounter difficulties passing the BBB, penetrating the tissue or uptaking in recipient cells when they are absorbed via nanotechnology for targeted therapy [80]. Luckily, exosomes can avoid these disadvantages and become ideal carriers. They are nano-sized, non-toxic, biocompatible and low immunogenicity [81,82]. Moreover, exosomes are stable drug delivery vehicles which can maintain their stability and activity during long-term storage [80]. Thus, several pharmaceutical substances and biomacromolecules have been successfully delivered based on exosomes for cancer treatment, encompassing small molecules, nucleic acids and proteins (Table 2) [83].

#### 3.1.1. Small Molecules

Many drugs with small molecules have been loaded into exosomes for cancers treatment, such as paclitaxel, doxorubicin, curcumin, etc. These drugs, carried by exosomes, can improve anticancer activity, drug targeting, drug delivery efficiency, and reduce drug toxicity and drug resistance, etc. Pascucci L. et al. delivered paclitaxel (PAC) by exosomes to treat pancreatic cancer, and its anti-proliferative activity significantly increased compared with the free drug in vitro [84,93]. Tian et al. loaded doxorubicin into exosomes for the treatment of breast cancer and found that the immunogenicity and toxicity of doxorubicin were reduced significantly [85]. Zhuang et al. found that curcumin-loaded exosomes exhibited strongly targeted ability in the brain, effectively inhibiting tumor growth in mouse models [86]. Wang et al. studied the anticancer effect of cisplatin-loaded exosomes, which inhibited the progression of hepatocellular carcinoma and prolonged mice survival [13]. Lessi et al. demonstrated that acridine orange encapsulated by exosomes was efficacious for the treatment of melanoma in vitro, with a prolonged drug delivery efficiency [87]. Ma et al. developed a novel method of using tumor cell-derived exosomes containing anti-cancer drugs to eliminate drug-resistant properties in tumor-repopulating cells (TRCs).

#### 3.1.2. Nucleic Acids

Exosomes can deliver nucleic acids such as miRNA and siRNA to therapeutic targets in cancer treatment [94]. miRNA and siRNA are unstable in circulation, easy to be eliminated and struggle to enter targeted cells. The transmission of miRNA and siRNA through exosomes can prevent their decomposition in vivo and achieve a better therapeutic effect [95]. Ohno et al. used exosomes as vectors to deliver steadily exogenous miRNA let-7a to breast cells, inhibiting tumor growth by reducing RAS and HMGA2 expression [88]. Munoz et al. loaded anti-miR-9 into exosomes and then delivered it to glioblastoma cells, which enhanced the therapeutic effects remarkably [89]. Shtam et al. loaded 98RAD51- and RAD52-siRNA into exosomes, and the stowage can be delivered steadily to fibro sarcoma cells, reducing the viability and proliferation of cancer cells [91]. In addition, the immunogenicity of siRNA can be overcome by loading siRNA into exosomes. Usman et al. found that the exosome loaded with antonym oligonucleotides has a significant inhibitory effect on breast cancer cells and has no immunogenicity in the body.

#### 3.1.3. Proteins

Exosomes can be used to transport large molecules at the same time, such as peptides and proteins. The desired protein can be transported into exosomes with minimum influence to its bioactivity. Furthermore, exosomes can protect protein from various enzymes and the immune system. Several proteins have been delivered for targeted therapy in cancer, such as catalase, superoxide dismutase, various antigens, proteasomes, transferrin and lactoferrin [96]. Lee et al. found that exosomes from transfected cells loaded with MHC-II and Trp2 significantly inhibited tumor growth [97]. Encapsulating proteins in exosomes such as survivor T34A would induce apoptosis in various pancreatic adenocarcinoma cell lines and increase their sensitivity to gemcitabine [92].

### 3.2. As Biomarkers for Cancer Diagnosis

Currently, liquid biopsy has emerged as a noninvasive and convenient approach for cancer diagnosis [98,99]. Compared to other sources of liquid biopsies, such as circulating tumor cells (CTCs) and circulating tumor DNA (ctDNA), exosomes show some superiorities in some special aspects. Firstly, exosome concentrations are higher than CTCs, thus avoiding procedures that require large amounts of blood or urine. Secondly, exosomes have high stability in the circulation, whereas ctDNA is rapidly degraded in the bloodstream [3,100,101]. Thirdly, exosomes can be gained from nearly all kinds of body fluids, including blood, urine, saliva, amniotic fluid, cerebrospinal fluids, bile, ascites, tears, breast milk and semen [102,103]. Accordingly, exosome-shuttled proteins and nucleic acids have been suggested as novel diagnostic and prognostic indicators for a variety of cancers [104] (Table 3).

#### 3.2.1. Nucleic Acids

The changes of exosomes components can reflect the physiological state of their original cells. Cancer cell-derived exosomes containing specific proteins and miRNAs are secreted into the TME and circulation [110]. Numerous studies have shown that nucleic acids are an integral part of exosomes, the most important of which are microRNAs (miRNAs), messenger RNAs (mRNAs), transfer RNAs (tRNAs) and long noncoding RNAs (lncRNAs) [76,111,112].

There are many types of miRNAs, and they are involved in the development of many types of cancers [97]. In studies, miRNAs can be used as common biomarkers or specific biomarkers for cancer diagnosis. In thyroid cancer, miR-146, miR-222, miR-31, miR-21, miR-181A-5p, miR-346, miR-34a-5p, miR-10a-5p, miR-5189-3p, miR-485-3p and miR-4433a-5p all served as biomarkers [113]. Among them, miR-5189-3p showed the best diagnostic effect. Through small RNA sequencing and comprehensive analysis, Samsonov et al. isolated exosomes from the plasma of patients with papillary thyroid cancer (PTC) and identified exosomal miRNAs as candidate biomarkers [113]. Jin et al. found that the expression levels of miR-21-5p, miR-1246, miR-1229-5p and miR-96-5p in the control group were lower than those of chemotherapy-sensitive patients, suggesting that miRNAs in exosomes could predict the chemical resistance of patients with colorectal cancer and were expected to become new targets for the treatment of drug resistance [105]. Evidence proved miR-21 is a very special miRNA, since it can be used as a biomarker for many types of cancers such as thyroid cancer, stomach cancer, liver cancer, colon cancer, pancreatic cancer, ovarian cancer, melanoma cancer and glioma cancer [8,94,95,106,114,115,116]. For example, MiR-21 could promote the proliferation and metastasis of HCC cells by inhibiting the expression of PTEN, PDCD4, RECK and HSULF-1 (human sulfatase-1), even making HCC cells resistant to chemotherapy. The level of blood exosomal miR-21 level in patients with HCC is significantly higher than those in healthy people. Elevated serum exosomal miR-21 level is often positively correlated with tumor development, thus exosomal miR-21 could be used as a potential diagnostic marker for HCC [115]. In addition, miRNAs can be also served as biomarkers in hematological tumors. miR-29 seemed to play a key role in hematological malignancies through the regulation of TCL1, MCL1 and DNA-methyltransferases. miR-223 was downregulated in CLL (chronic lymphocytic leukemia)-derived exosomes in contrast to the healthy controls [117].

In addition to miRNAs, exosomal lncRNAs from cancer patients have been defined as novel tumor biomarkers [81]. In different types of cancers, specific exosomal lncRNAs reflect different pathophysiological states, revealing the clinical status of cancer [118]. In a review of Sun et al., exosomal lncRNA CRNDE-h was identified in serum of colorectal cancer (CRC) patients. Therefore, lncRNA is a potential as diagnostic and therapeutic tool for CRC [119]. Du et al. analyzed the expression and function of lncRNA PCAT-14 in hepatocellular carcinoma, which was overexpressed in HCC patients [107].

Meanwhile, circRNAs are specific biomarkers for certain cancers [120]. The relationship between exosomal circRNA and tumor diagnosis was mainly concentrated in the diagnosis of lung cancer and breast cancer [121]. Zhu et al. determined that the presence of hsa-circ-0013958 in the plasma of lung cancer patients was positively correlated with tumor metastasis, and it could be used as a biomarker for functional diagnosis and prognosis of lung cancer [122]. Yang et al. found that circ-PTK2 was upregulated in CRC tissues and was associated with poor tumor growth, metastasis and overall survival [108]. Li et al. found that circ RNAs has-circ-002059 and has-circ-104916 were significantly downregulated in plasma and gastric cancer tissue, suggesting the potential of these circ RNAs as biomarkers for stable diagnosis of gastric cancer [84].

#### 3.2.2. Proteins

Exosomes contain a variety of proteins, such as the annexin, flotillin, Hsp70, Hsp90, CD9, CD37, CD53, CD63, Alix, Tsg101, etc., which reflect the status of parental cells [96]. Exosomal proteins from cancer cells are gradually becoming ideal biomarkers for cancer monitoring and efficacy evaluation. Exosomal proteins possess unique features over traditional serological markers. First, exosomal proteins have a higher sensitivity compared with proteins directly detected in blood. Second, exosomal proteins have a higher specificity over secretory proteins. Third, exosomal proteins are highly stable. Exosomal proteins are protected from external proteases and other enzymes by the lipid bilayer, and phosphorylation proteins can be separated from exosomal samples frozen for five years [123]. Thus, in vivo detection of exosomes is highly sensitive and conducive to diagnosis of early-stage cancer.

As biomarkers of cancer, exosomal proteins are mainly used in gastric cancer, ovarian cancer, breast cancer and melanoma. For example, in gastric cancer (GC), several exosomal proteins have been reported to be associated with the progression of GC, including TGF-β1, human gastrokine 1, Cag A and apolipoprotein E. Yoon et al. found that the level of GKN1 in GC patients was significantly lower than that in healthy people. In addition, serum GKN1 levels could distinguish GC patients from those with HCC and colorectal cancer. Therefore, serum GKN1 can be used as a GC specific diagnostic marker [9]. In breast cancer, some exosomal proteins are used as biomarkers, including heat shock protein 70 (HSP70), platelet-reactive protein 1 (TSP1), lactate dehydrogenase C4 (LDH-C4), exo-Anx2 and Integrin protein α6. Cen et al. demonstrated that platelet-reactive protein 1 (TSP1) was extraordinarily expressed in exosomes derived from BC cells, which would be a biomarker for BC progression [109].

Despite the advance in the search for diagnostic and prognostic markers, none of the mentioned that EV-associated biomarkers have been approved by national or international agencies. The U.S. Food and Drug Administration (FDA) controls the approval of new biomedical tests and device tests [124]. The potential of using exosomal markers for clinical diagnosis needs to be further investigated in depth [125].

### 3.3. As Therapeutic Target

Targeted therapy is a treatment that established cancerous sites at the cellular and molecular level [126]. More and more evidence demonstrates that exosomes play a role in cellular processes that influence tumorigenesis, progression and metastasis. Thus, apart from being drug carriers and biomarkers, exosomes have the potential ability to be anticancer targets [127].

As therapeutic targets, there are two major strategies to mediate the process of cancer cells development: inhibiting the release of cancer cell-derived exosomes and blocking the uptake of specific exosomes with receptor cells [116]. For example, Guan et al. reported proton-pump inhibitors (PPIs) could inhibit the release of exosomes from gastric cancer (GC) cells, suggesting PPIs might be of potential value as a therapeutic tool for GC treatment [94]. Lan et al. demonstrated that another exosome inhibitor GW4869 could reduce the survival rate of pancreatic cancer (PC) cells [116]. Nakamura et al. also indicated that GW4869 was an inhibitor that impaired exosomes secretion from 293T cells [127]. These findings implicated a potential utility of exosome inhibitors as novel adjunct therapeutic strategies for advanced cancer treatment [128].

Another effective strategy for exosome-targeted therapy is to inhibit the uptake of exosomes with recipient cells. Heparan sulfate proteoglycans (HSPGs) are internalizing receptors of cancer-derived exosomes which could significantly attenuate exosomes uptake [129]. In addition, Reg3β, a lectin binding to the surface of exosomes, was released by normal pancreatic tissue surrounding tumors. It could block the uptake of exosomes released by tumor cells both in vitro and vivo, further inhibiting cancer cell migration and metabolic changes. This approach might be a strategy for treating pancreatic cancer [18].

## 4. The Multiple Performance of Different Sources of Exosomes in Cancers

The roles of exosomes in disease treatment are relevant to their original parental cells [130]. Exosomes from mesenchymal stem cells (MSCs) and cancer cells are widespread in the therapeutic process against cancers (Table 4).

### 4.1. MSCs-Derived Exosomes

**Figure 3:**
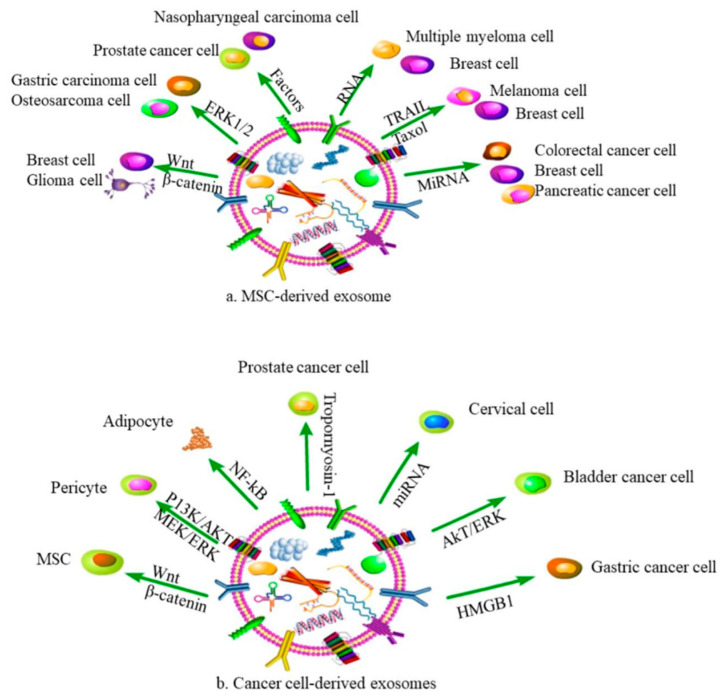
Effects of exosomes from different sources on cancers. (**a**,**b**) respectively show that exosomes from MSCs and cancer cells affect various cancers processes through different inclusions.

MSCs, with the capability of self-renewal and multidirectional differentiation, are the ideal producer of exosomes. As the products of MSCs, exosomes can regulate the proliferation, angiogenesis and migration of cancer cells. The expression changes of exosomal components are also related to the occurrence of cancers, such as proteins, mRNA, miRNA, lncRNA and so on (Figure 3a). Xing et al. indicated that MSCs-derived exosomes led to the upregulation of miR-106a-5p and tumor progression in triple-negative breast cancer [131]. Deng et al. reported that MSCs-derived exosomes promoted multiple myeloma tumorigenesis through lncRNA LINC00461, which was highly expressed in patients [132].

In addition to regulating the expression of exosomal components, MSCs-exosomes could also mediate the development of cancer by several signaling pathways. Wnt/b-catenin signaling pathway was activated after MSCs-derived exosomes treatment in MCF7 cells, which promoted the migration of breast cancer cells [133]. There was another study showing that MSCs-derived exosomes enriched miR-208a, promoting osteosarcoma cell viability, clonogenicity and migration by activating the ERK1/2 pathway [134]. Shi et al. observed that MSCs-derived exosomes enhanced the growth and migration of nasopharyngeal carcinoma (NPC) via activating the fibroblast growth factor (FGF) signaling pathway [141]. Zhu et al. found that exosomes secreted by MSCs promoted tumor growth through the activation of EPK1/2 and MAPK pathway [135]. Che et al. investigated the effect of MSCs-derived exosomal miR-143 on prostate cancer. Their results showed that the overexpression of miR-143 could inhibit proliferation and the migration of prostate cancer cells through downregulating TFF3 [138]. Xu et al. confirmed that MSCs-derived exosomes loaded miR-133b repressed glioma cell proliferation, invasion and migration by silencing EZH2 and inhibiting the Wnt/β-catenin signaling pathway [135].

Considering the efficient biocompatibility and relatively acceptable tissue tolerance, MSCs-derived exosomes were also used as vehicles to deliver different small molecules, natural compounds or chemotherapeutics in tumor treatment. Melzer et al. proposed that taxol-loaded MSCs-derived exosomes inhibited breast cell growth and metastasis [130]. Shamili et al. indicated TRAIL-loaded MSCs-derived inhibited melanoma progression due to cell necrosis [137]. Vakhshiteh et al. investigated the function of miR-34a-encapsulated by MSCs-derived exosomes in breast carcinoma, which suppressed tumor metastasis and invasion [138]. In addition, MSCs-derived exosomes carried by miR-124 could reduce the invasion and migration of pancreatic cancer [139]. Liang et al. observed that miR-499 loaded with MSCs-derived exosomes suppressed cell proliferation in endometrial cancer cells [140]. Li et al. proposed that that miR-3940-5p encapsulated MSCs-derived exosomes inhibited invasion and metastasis of colorectal cancer cells [152].

### 4.2. Cancer Cell-Derived Exosomes

Carcinoma-associated fibroblasts (CAFs) are promising therapeutic targets to counteract cancer progression, (Figure 3b). Yeon et al. observed that exosomes secreted by cancer cells significantly influenced the expressions of CFAs via regulating FSP-1 and α-SMA [145]. This was in accordance with a previous study that found gastric cancer cell-derived exosomes enhanced pericytes proliferation, migration and transition to CFAs via activating PI3K/AKT and MEK/ERK pathways [146]. It was suggested that tumor-derived exosomes could promote tumor malignancy by secreting tumor-promoting factors and activating the TGF β receptor-mediated signaling pathway [144]. Wang et al. explored the role of hepatocellular carcinoma (HCC)-derived exosomes, which enhanced tumor growth through activating NF-κB signaling pathway and converting adipocytes into tumor-promoting cells [147]. Dai et al. demonstrated that exosomal miR-183 derived from prostate cancer cells facilitated tumor proliferation, migration and invasion via downregulating tropomyosin-1 [148]. You et al. showed that cervical cancer cell-derived exosomes, enriched in miR-663b, promoted cervical cancer tumor metastasis via activating EMT signaling pathway [149]. Yang et al. demonstrated that exosomes derived from bladder cancer cells inhibited tumor cells apoptosis via activating Akt and ERK pathway [150]. Zhang et al. found gastric cancer cell-derived exosomes promoted tumor migration through inducing the autophagy of neutrophils [151].

Furthermore, cancer cell-derived exosomes could mediate tumor formation and development by interfering with MSCs. Ma et al. found that glioma cell-derived exosomes promoted the proliferation, invasion and migration of MSCs by activating glycolysis [142]. Gastric cancer cell-derived exosomes (GC-Exos) affected the immunomodulation function of MSCs through the NF-κB signaling pathway [143]. In addition, GC-Exos induced the differentiation of MSCs to carcinoma-associated fibroblasts [144].

## 5. Conclusions

**Figure 4:**
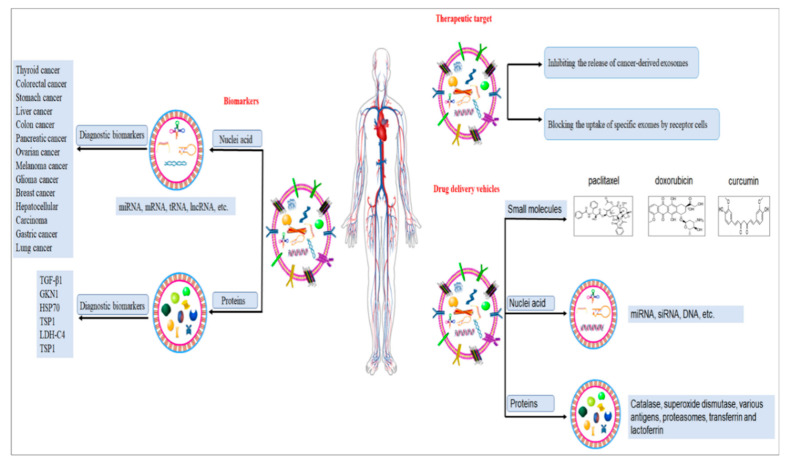
The applications of exosomes in cancers.

The current research on exosomes has provided new avenues for optimized cancer diagnosis and therapy (Figure 4). Obtained data indicate that exosomes are involved in cancer development by regulating some intermediate regulatory factors. They have gained extensive attention in cancer research due to their multifaceted roles, such as reprogramming tumor behaviors and remodeling the TME [153]. As carriers, exosomes may represent a new class of drug delivery system due to their ability to cross biological barriers with little or no safety concerns associated with therapeutics, including drug toxicity, immune responses, biodistribution and targeted delivery. As biomarkers, the exosome can provide abundant, stable, sensitive and specific biological information and is a liquid biopsy specimen with high application value. Furthermore, exosomes are emerging as valuable therapeutic targets closely aligned with the development of precision medicine. However, many problems still need to be resolved. In the TME, the way that exosomes influence distant cell interactions within the tumor microenvironment has not yet been fully understood. In therapy, determining which sources of exosomes are safe and bio-compatible for drug delivery system is still an obscure problem; the exosome drug-loading methods and targeted modification technology need to be further improved in clinical application; in diagnosis, the use of exosomes as cancer biomarkers is technically limited by their size, heterogeneity and labeling; it is difficult to determine which RNAs and proteins are suitable for use as tumor inhibitors and cancer biomarkers; and the identification of clinically relevant exosomes among numerous other populations of exosomes secreted by almost all body cells is still a challenge, mainly due to the lack of adequately sensitive and fast analysis platforms. In addition, the cost effectiveness, rapid isolation and purification of exosomes remain the main obstacles for their use in clinical application. The exosome concepts are significantly correlated with origin cells and source, so the role of exosomes from different sources also needs further study. We also need to exploit more tools to uncover the molecular nature of exosomes and the categories of exosomes containing supportive tumor molecules. Moreover, the majority of experiments are carried out in vitro and ultimately in vivo animal models; therefore, the safety, specificity and efficiency of this system in clinical trials still remains elusive. Thus, there is still an unmet clinical need for improved detection and therapy strategies for cancer treatment [154,155]. Nonetheless, it is believed that, with the tremendous support of proteomics, genomics, nanotechnologies, high-throughput sequencing technology and bioinformatics data analysis technologies, exosomes will promise an unparalleled prospect in the diagnosis and therapy of cancers in the coming years.

## Figures and Tables

**Table 1:** Exosome-mediated regulation of lymphoid functions in cancer.

Origin of Exosomes	Functional Molecules	Target Cells/Molecules	Response in the Target Cells/Molecules	References
Intestinal epithelial cells	MHC class I and II	T cells	Exosomal MHC complexes activated T cells by enhancing antigen presentation of DC cells	[32]
CT26 mouse colon carcinoma cells (H-2^d^) and B16-F1 mouse melanoma cells (H-2^b^)	HSP 70	T cells	HSP70-enriched exosomes induced Th1 immune responses, resulting in eliminating cancer cells in vivo	[33]
Glioma cells	MAGE-1	T cells	MAGE-1 activated T cells to become glioma specialized CTL	[34]
Glioma cells	ICAM-1	T cells	ICAM-1 activated T cells to become glioma-specialized CTL	[34]
Human prostate cancer cell line	FasL	CD8+T cells	Exosomes-enriched Fas ligand mediated CD8^+^ T cells apoptosis	[35]
Colorectal cancer	FasL	T cells	Exosomes carrying Fas ligand induced T cells apoptosis	[36]
Melanoma, non-small-cell lung cancer and renal cancer	PD-L1	T cells	Exosomal PD-L1 bound with its receptor influenced on T cells and accelerated tumor growth	[37]
PBMC of cancer patients	TGF-β IL-10	T cells	TGF-β and IL-10 promoted the generation of CD4^+^ CD25^+^ Foxp3^+^ Tregs, activated STAT3 and increased the number of phosphorylated Smad 2/3	[38]
Mycoplasma-infected tumor cells	IFN-c IL-10	B cells	Exosomes activated splenic B cells and induced splenocytes cytokine IFN-c and IL-10 production	[39]
Pancreatic adenocarcinoma cells	immunoglobulins	B cells	Exosomal TAAs bound circulating autoantibodies and inhibited cell-mediated cytotoxicity	[40]
Chronic B lymphocytic leukemia	gp350	B cells	gp350 bound to the CD154 receptor on B cells and rendered B cells immunogenic	[41]
Esophageal carcinoma	MMP9	B cells	Caner-derived exosomes carried MMP9 to activate TGF-β in B cells to facilitate Breg differentiation	[42]
Acute myeloid leukemia	TGF-β	NK cells	Exosomal TGFβ decreased NK cells cytotoxicity and downregulated the expression of NKG2D	[43]
Lung carcinoma cells	miR-23a	NK cells	Exosomal miR-23a targeted the expression of CD107a in NK cells and produced immunosuppression	[44]
Advanced pleural malignant mesothelioma	IL-2	NK cells	Exosomes promoted the differentiation and proliferation of NK cells by increasing the secretion of IL-2	[45]
EGCG-treated breast cancer cells	miR-16	M2	Exosomal miR-16 induced M1 polariation	[46]
Rectal cell carcinoma	miR-203	M2	Exosomal miR-203 promoted the expression of M2 markers	[47]
Breast cancer	glycoprotein 130	M2	Exosomal glycoprotein 130 enhanced STAT3 activation and induced M2 polarization	[48]
Oral squamous cell carcinoma	THBS1	M1	Exosomal THBS1 induced M1 polariation	[49]
Colorectal cancer	miR-21	M1	Exosomal miR-21 induced M1 polarization and the secretion of IL-6	[50]

**Table 2:** The applications of exosomes as drug carriers.

Cancer Types	Loading Drug	Outcome	References
Hepatocellular carcinoma	cisplatin	Cisplatin-loaded exosomes inhibited the progression of hepatocellular carcinoma	[13]
Pancreatic cancer	paclitaxel	The anti-proliferative activity of paclitaxel increased compared with the free drug in pancreatic cancer	[84]
Breast cancer	doxorubicin	The immunogenicity and toxicity of doxorubicin increased significantly in breast cancer	[85]
Brain tumor	curcumin	Curcumin-loaded exosomes exhibited strongly targeted ability in the brain, effectively inhibiting tumor growth in vivo	[86]
Melanoma	acridine orange	The acridine orange encapsulated by exosomes prolonged drug delivery efficiency in melanoma	[87]
Breast tumor	miRNA let-7a	MiRNA let-7a loaded by exosomes could inhibit breast tumor growth	[88]
Glioblastoma	anti-miR-9	The anti-MiR-9 loaded by exosomes could sensitize the glioblastoma multiforme cells to chemotherapy drugs	[89]
Sarcomas	TGFβ1 siRNA	The TGFβ1 siRNA loaded by exosomes could inhibit the growth of mouse sarcomas both in vitro and in vivo	[90]
Fibro sarcoma	98RAD51-siRNA, RAD52-siRNANA	The RAD52-siRNA loaded by exosomes could reduce the viability and proliferation of cancer cells	[91]
Pancreatic cancer	survivor -T34A	The survivor -T34A loaded by exosomes could induce apoptosis in various pancreatic adenocarcinoma cell lines and increase their sensitivity to gemcitabine	[92]

**Table 3:** The applications of exosomes as biomarkers.

Cancer Types	Biomarker	Outcome	References
Gastric cancer	has-circ-002059, has-circ- 104916	The circ RNAs has-circ-002059 and has-circ-104916 were significantly downregulated in plasma and gastric cancer tissue	[84]
Gastric cancer	TGF-β1, Cag A, Apolipo- protein E, human gastrokine 1	The level of GKN1 in GC patients was significantly lower than that in healthy people	[94]
Colorectal cancer	miR-21-5p, miR-1246, miR-1229-5p, miR-96-5p	The expression levels of miR-21-5p, miR-1246, miR-1229-5p and miR-96-5p in healthy people were lower than those of chemotherapy-sensitive patients	[105]
Hepatocellular carcinoma	miR-21	The level of blood exosomal miR-21 levels in patients with hepatocellular carcinoma were significantly higher than those in healthy people	[106]
Hepatocellular carcinoma	lncRNA PCAT-14	The level of blood exosomal lncRNA PCAT-14 was upregulated in hepatocellular carcinoma patients	[107]
Colorectal cancer	circ-PTK2	The circ-PTK2 was upregulated in tissues of colorectal cancer patients	[108]
Breast cancer	thrombocyte reactive protein 1	The thrombocyte reactive protein 1 was overexpressed in breast cancer	[109]

**Table 4:** The roles of exosomes from different sources in cancers.

Origin of Exosomes	Outcome	References
MSCs-derived exosomes	Promoted TNBC progression	[131]
	Promoted proliferation and suppressed apoptosis of MM cells	[132]
	Promoted migration of breast cancer cells	[133]
	Promoted osteosarcoma cells proliferation, migration and invasion	[134]
	Enhanced the growth and migration of NPC cells	[135]
	Favored gastric carcinoma tumor growth and angiogenesis	[136]
	Suppressed cells proliferation, migration and invasion of glioma	[135]
	Inhibited breast tumor growth and reduced distant organ metastases	[130]
	Inhibited melanoma cells growth	[137]
	Decreased the migration and invasion of breast carcinoma cells	[138]
	Induced pancreatic cells apoptosis and reduced the invasion, migration of cells	[139]
	Inhibited endometrial cancer growth and angiogenesis	[140]
	Inhibited invasion as well as growth and metastasis of colorectal cancer cells	[141]
	Promoted the growth, migration and invasion of HBMSCs	[142]
	Enhanced the ability of MSCs to activate immune cells and supported tumor growth	[143]
	Induced the differentiation of MSCs to CAFs	[144]
	Suppressed proliferation, migration, invasion and enhanced apoptosis of PCa cells	[64]
Cancer cell-derived exosomes	Influenced the expressions of CFAs	[145]
	Enhanced pericytes proliferation and migration	[146]
	Enhanced hepatocellular carcinoma tumor growth	[147]
	Facilitated proliferation, migration and invasion of prostate cancer cells	[148]
	Promoted cervical cancer tumor metastasis of cervical cancer cells	[149]
	Inhibited bladder cancer tumor cells apoptosis	[150]
	Promoted migration and proliferation of gastric cancer cells	[151]

## Abbreviations

TME	Tumor microenvironment
MSCs	Mesenchymal stem cells
CAFs	Cancer-associated fibroblasts
CSCs	cancer stem cells
NK	natural killer
TEXs	Tumor-derived exosomes
TAAs	Tumor-associated antigens
HCC	human hepatocellular carcinoma
ECA	esophageal carcinoma tissue
KIRs	killer immunoglobulin receptors
NCRs	natural cytotoxicity receptors
AML	acute myelogenous leukaemia
TAMs	Tumor-associated macrophages
EGCG	epigallocatechin gallate
DDS	drug delivery systems
PAC	paclitaxel
TRCs	tumor-repopulating cells
CTCs	circulating tumor cells
PTC	papillary thyroid cancer
CRC	colorectal cancer
HSP70	heat shock protein 70
TSP1	platelet-reactive protein 1
LDH-C4	lactate dehydrogenase C4
FDA	Food and Drug Administration
PPIs	proton-pump inhibitors
GC	gastric cancer
PC	pancreatic cancer
HSPGs	Heparan sulfate proteoglycans
NPC	nasopharyngeal carcinoma
FGF	fibroblast growth factor
GC-Exos	Gastric cancer cell-derived exosomes
CAFs	Carcinoma-associated fibroblasts
